# β-Si_3_N_4_ Microcrystals Prepared by Carbothermal Reduction-Nitridation of Quartz

**DOI:** 10.3390/ma12213622

**Published:** 2019-11-04

**Authors:** Meng Zhang, Zhi Chen, Juntong Huang, Saifang Huang, Zhihui Hu, Zhijun Feng, Qingming Xiong, Xibao Li

**Affiliations:** 1 School of Materials Science and Engineering, Nanchang Hangkong University, Nanchang 330063, Jiangxi Province, China; zhangmeng4747@163.com (M.Z.); chenzhi_2018@126.com (Z.C.); Huzhihui94@163.com (Z.H.); fengzhijun@nchu.edu.cn (Z.F.); xiongqingming123@163.com (Q.X.); 2 Department of Chemical and Materials Engineering, University of Auckland, Private Bag 92019, Auckland 1142, New Zealand; shua084@aucklanduni.ac.nz

**Keywords:** β-Si_3_N_4_ microcrystals, Fe_3_Si archimedean solids, quartz, carbothermal reduction nitridation, seeding

## Abstract

Single phase β-Si_3_N_4_ with microcrystals was synthesized via carbothermal reduction-nitridation (CRN) of quartz and carbon coke powder as starting materials. The effects of reaction parameters, i.e., heating temperature, holding time, C/SiO_2_ ratio, Fe_2_O_3_ additive and β-Si_3_N_4_ seeds on the phase transformation and morphology of products were investigated and discussed. Rather than receiving a mixture of both α- and β- phases of Si_3_N_4_ in the products, we synthesized powders of β-Si_3_N_4_ single polymorph in this work. The mechanism for the CRN synthesis of β-Si_3_N_4_ from quartz and the formation mechanism of Fe_3_Si droplets were discussed. We also firstly reported the formation of Fe_3_Si Archimedean solids from a CRN process where Fe_2_O_3_ was introduced as additive. Comparing to the gear-like short columnar morphology observed in samples without β-Si_3_N_4_ seeding, the addition of β-Si_3_N_4_ seeds led to an elongated morphology of final products and much finer widths. In addition, the β-Si_3_N_4_ microcrystals exhibited a violet‒blue spectral emission range, which could be highly valuable for their future potential optoelectronic applications.

## 1. Introduction

Silicon nitride (Si_3_N_4_) is an important high temperature structural material because of its excellent properties, including high strength, high decomposition temperature (1900 °C), good resistance to oxidation, thermal shock, corrosive environments, which have been investigated extensively over the past three decades [1,2,3,4].

The most prevalent methods for preparing Si_3_N_4_ powders include direct nitridation method, carbothermal reduction-nitridation (CRN) method and thermal decomposition method [5,6,7]. By CRN method, Si_3_N_4_ powders or columnar grains with excellent size distribution and physical properties could be synthesized and used as thermal conductive fillers or commercial applications for manufacturing engineering devices [8,9]. For example, Karakus et al. [9]. synthesized α-Si_3_N_4_ powders by CRN of synthetic silica and activated charcoal at 1470 °C, and then used the obtained α-Si_3_N_4_ powders as the raw materials to prepare the Si_3_N_4_ ceramic by a pressureless sintering method. Comparing the results with commercial Si_3_N_4_ powders, the resultant Si_3_N_4_ powders by CRN method indicated a similar or ever better density and β-phase conversion by the pressureless sintering. Yin et al. prepared ZrN–Si_3_N_4_ composite powders from natural zircon and quartz via CRN reaction at temperatures below 1600 °C [10]. Similarly, Arik prepared Si_3_N_4_ powders by CRN from diatomite with C/SiO_2_ molar ratio 4 at 1400 °C for 16 h [11]. Thus, it is feasible to prepare Si_3_N_4_ with quartz and carbon black. On the other hand, CRN method takes advantage from low-cost starting materials [8,9]. The high cost of raw materials is a primary limitation for large scale production of Si_3_N_4_ powders. Through the CRN method, it is possible to synthesize Si_3_N_4_ powders from low-cost quartz with abundant reserves in the world. It was reported that the same problem for the massive production of SiC powders was overcome by this way, and the product was much finer for achieving excellent flexural strength [12].

Grain size and shape of Si_3_N_4_ powders can influence some properties of Si_3_N_4_-based products such as varying electrical and optical properties and mechanical properties [13,14]. Apart from Si_3_N_4_ seeds, Fe and its oxides have been used as additives to change the morphology of a product or to promote nitriding process [15,16,17,18]. However, to the best of our knowledge, the crystal microstructure/morphology of Fe-containing compounds formed in the nitridation process while Fe or iron oxide being used as additive was rarely reported.

In this study, quartz and carbon coke powders were selected as raw materials to prepare Si_3_N_4_ powders via CRN method. The influence of temperature, holding times, C/SiO_2_ molar ratio, additive amount of Fe_2_O_3_ and β-Si_3_N_4_ seeds were studied on the phase transformation and morphology of products. Faceted Fe_3_Si Archimedean solids, a novel morphology of the iron silicide, were observed firstly which have the potential application in spintronics devices [19]. The formation mechanism of products from the nitridation reaction was discussed and photoluminescence (PL) properties of samples were also detected.

## 2. Experimental

### 2.1. Materials

**Figure 1:**
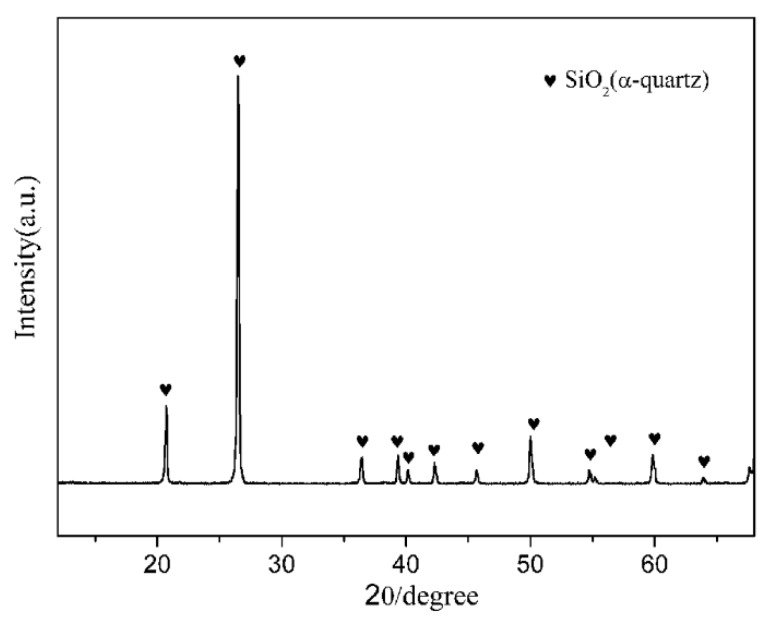
X-ray diffraction (XRD) pattern of natural quartz.

Natural quartz powders (granularity ≤ 400 mesh, chemical composition (wt.%): SiO_2_: 97.8, Al_2_O_3_: 0.63, Fe_2_O_3_: 0.13, CaO: 0.08, K_2_O: 0.05, others: 1.31), and coke powders (granularity ≤ 200 mesh, carbon content = 88%) were used as the main starting raw materials. The crystalline phase of the natural quartz powders was hexagonal α-quartz (Figure 1). Fe_2_O_3_ (A.R. grade, Sinopharm Chemical Reagent Beijing Co., Ltd., Beijing, China) and Si_3_N_4_ (A.R. grade, ~800 nm, Shanghai Pantian powder material Co., Ltd., Shanghai, China) were used as additives. The starting compositions of all the samples are listed in Table 1.

### 2.2. Methods

The starting materials were ball-milled together for 2 h. Then, 2 g of the mixed powders were die-pressed under 20 MPa into a specimen of 10 mm in diameter. The specimens were placed in a graphite crucible and heated in flow nitrogen (purity 99.999%) in the reaction chamber of a tube furnace at temperatures in the range of 1450–1600 °C. A two stage heating schedule was used, i.e., heating up from ambient temperature to 1000 °C at 10 °C·min^−1^, then raising to the final temperature at 5 °C·min^−1^ and held for 3 h. The final temperatures were set as 1470 °C, 1500 °C, 1530 °C, 1550 °C, 1570 °C, and 1600 °C, respectively. In addition, in order to study the effect of holding time to the CRNed product, S1 sample was synthesized at 1600 °C and held for 1 h, 2 h, and 3 h, respectively. The fired samples were furnace-cooled to room temperature, and then reheated in air at 700 °C for 2 h to remove the residual carbon.

### 2.3. Characterization

The crystalline phases of the synthesized products were identified by X-ray diffraction (XRD; D8 Advance diffractometer, Bruker, Rheinstetten, Germany), using Cu Kα_1_ radiation (λ = 1.5406 Å) with a step of 0.02° (2θ) and a scanning rate of 4° min^−1^. The microstructures and morphologies of the products were observed by scanning electron microscopy (SEM; JEM-6460LV microscope, JEOL, Tokyo, Japan) and high-resolution transmission electron microscopy (HRTEM, JEM2010, JEOL, Tokyo, Japan). The energy dispersive X-ray spectroscopies (EDS) linked with the SEM and the HRTEM were employed to assist the micro-area chemical analysis of the products. FT-IR spectra were collected at room temperature using a FT-IR Spectrometer (FT/IR-4000 JASCO, Tokyo, Japan) equipped with a Michelson 28° interferometer with corner-cube mirrors, covering a range between 250,000 and 5 cm^−1^. Photoluminescence emission (PL) spectra were measured by F-7000 fluorescence spectrophotometer (Eppendorf, Shanghai, China) with a photomultiplier tube functioning at 700 V, and a 150 W Xe lamp as the excitation source.

## 3. Results and Discussion

### 3.1. Influence of Synthetic Schedule on the Phase Composition and Morphology of Products

#### 3.1.1. Influence of Temperature on the Phase Composition and Morphology of Products

**Figure 2:**
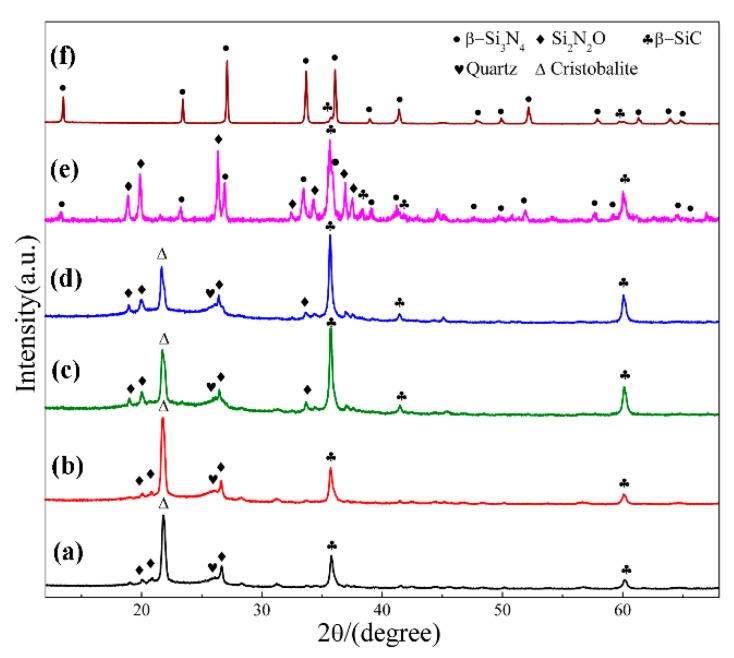
XRD patterns of sample S1 nitrided at different temperatures for 3 h: (**a**) 1470 °C; (**b**) 1500 °C; (**c**) 1530 °C; (**d**) 1550 °C; (**e**) 1570 °C; (**f**) 1600 °C.

Figure 2 shows XRD patterns of sample S1 nitrided at different temperatures for 3 h. As can be seen, cristobalite was the dominant phase at 1470 °C, which was phase transition from quartz. In additional, some SiC, Si_2_N_2_O and residual quartz were present in the products (Figure 2a). With nitriding temperature being gradually elevated from 1470 °C to 1550 °C, intensity of β-SiC and Si_2_N_2_O diffraction peaks increased and that of cristobalite decreased (Figure 2a–d). After further increasing the temperature to 1570 °C, β-Si_3_N_4_ had just emerged while its diffraction intensity was much lower than that of β-SiC and Si_2_N_2_O; in the meantime, the peaks of cristobalite disappeared, and no other silica phases were detected (Figure 2e). By applying a higher temperature of 1600 °C, β-Si_3_N_4_ was formed in a relatively high purity (Figure 2f). The intensity of the secondary phase β-SiC was very weak. The β-Si_3_N_4_ synthesized at 1570 & 1600 °C (Figure 2e,f) in S1 crystallized as a hexagonal structure and their lattice parameters and cell volumes are listed in Table 2. They were increased from 1570 to 1600 °C. The weight fraction of each phase in S1 at 1570 & 1600 °C is shown Table 3, the weight fraction was remarkable increased from 32.30 to 91.52 with the increased of temperature to 1600 °C. Upon the above observation, it is clear that in this work, the effect of temperature on the phase formation from quartz by CRN is very significant. For the holding period of 3 h, the temperature 1570 °C was critical for the transformation of cristobalite into Si_3_N_4_, whereas it cannot be formed at a lightly lower temperature of 1550 °C. Moreover, a temperature of 1600 °C, 30 °C higher than 1570 °C contributed to the formation of high-purity Si_3_N_4_ powders.

**Figure 3:**
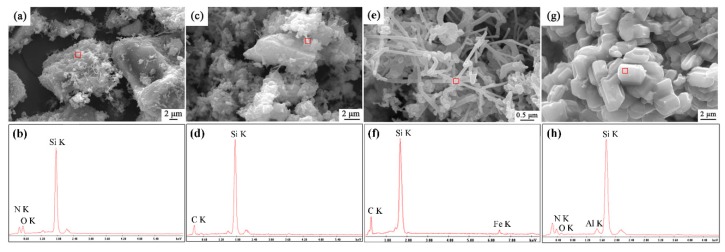
SEM images and X-ray spectroscopies (EDS) patterns of sample S1 nitrided at different temperatures for 3 h: (**a**,**b**)1500 °C, (**c**,**d**) 1550 °C, (**e**,**f**) 1570 °C (**g**,**h**) 1600 °C.

Morphological variation of sample S1 after being nitrided at different nitriding temperatures was investigated by SEM observation, and the elemental composition was performed by EDS analysis (Figure 3). There were many independent SiO_2_ particles with size of 20 μm, covered by some Si_2_N_2_O and SiC fibers at temperatures of 1500–1550 °C, confirmed by EDS (Figure 3a–d). Some Fe element in the fibers was detected in the EDS, which should be from original quartz raw material (Figure 3e,f). In the sample synthesized at 1600 °C, short columnar grains with diameters of about 2 μm were dominant throughout the sample, which is the typical morphology of β-phase Si_3_N_4_. The trace amounts of Al and O was detected in the grains; this might come from impurity of original quartz or ball milling.

#### 3.1.2. Influence of Holding Time on the Phase Composition of Products

**Figure 4:**
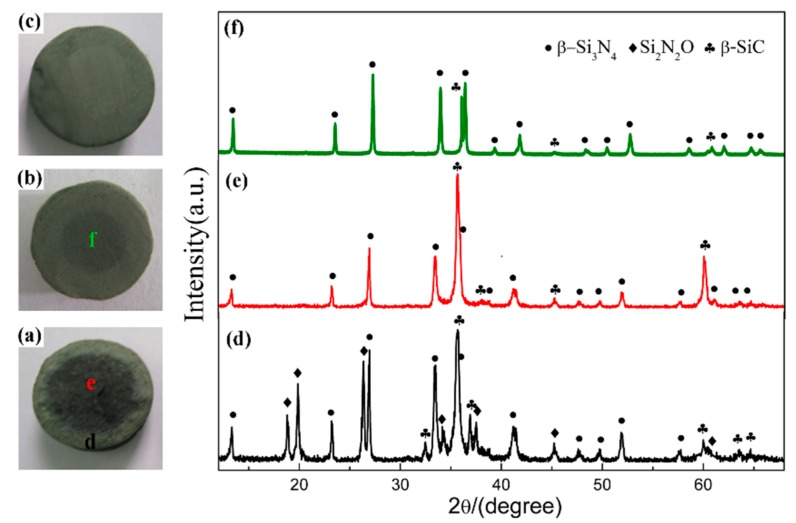
Digital photos of sample S1 nitrided at 1600 °C with different holding time: (**a**) 1 h, (**b**) 2 h, (**c**) 3 h; and XRD patterns of sample S1 in different regions: (**d**) exterior region and (**e**) interior region of pellet reacted for 1 h, (**f**) interior region of the pellet reacted for 2 h.

In order to elucidate the effect of holding time, the samples synthesized at 1600 °C for different holding times were compared. Figure 4a–c present the digital photos of three samples, which clearly demonstrated the degree of nitridation from the change in the nitridized area. As seen from the digital photos, 2 h was insufficient for CRN of quartz at 1600 °C. There were two different zones on the cross section of products (Figure 4a,b). The interior portion of pellets had a darker color while the exterior part was greenish gray. Interior regions of product narrowed down when holding time was 2 h and disappeared when 3 h. A longer holding time of 3 h enabled the complete nitridation of the entire pellet with diameter of <10 mm. The XRD patterns of them are depicted in Figure 4d,f. It is revealed that phase compositions were Si_3_N_4_, SiC, and Si_2_N_2_O for exterior region as well as SiC and Si_3_N_4_ for interior region with holding time of 1 h (Figure 4d,e). The diffraction peaks of SiC in interior region were evidently weakened with increasing the holding time to 2 h (Figure 4f) even almost disappeared after 3 h (Figure 2f). Since the permeation of N_2_ in samples was from outside to inside, the external N_2_ of samples was more abundant than that of internal. Increasing holding time allowed complete penetration of N_2_ into the inner pellets, leading to the fully nitridation of the entire pellets.

#### 3.1.3. The CRN Mechanism of Quartz

Based on the results described above and literatures reported previously [20,21], it is generally accepted that the CRN reaction of quartz would happen through several steps. The reduction of quartz into SiO, via the pathways as shown by Equation (1) (*∆G* is the Gibbs free energy of reaction, and T is the temperature), is the first but a critical step, which enables the further reduction process.

SiO_2_(s) + C(s) → SiO(g) + CO(g), *ΔG* = 665.578–0.33T kJ/mol (1)

The as-reduced SiO (g) is then nitrided into silicon nitride phases by carbon through the Equation (2) [22,23].

3SiO(g) + 3C(s) + 2N_2_(g) → Si_3_N_4_(s) + 3CO(g), *ΔG* = −830.44 + 0.35T kJ/mol (2)

At a temperature of 1550 °C or lower, intermediate phases Si_2_N_2_O and SiC were preferably formed through Equations (3) and (4), respectively [22,24]. It suggests that under the subcritical temperatures (1470 to 1550 °C), the reaction process was dominated by the reductive atmosphere/conditions while the nitridation processes were less thermodynamically preferred.

2SiO_2_(s) + 3C(s) + N_2_(g) → Si_2_N_2_O(s) + 3CO(g), *ΔG* = 572.54–0.32T kJ/mol (3)

SiO(g) + 2C(s) → SiC(s) + CO(g), *ΔG* = −78.60 + 0.01 T kJ/mol (4)

**Figure 5:**
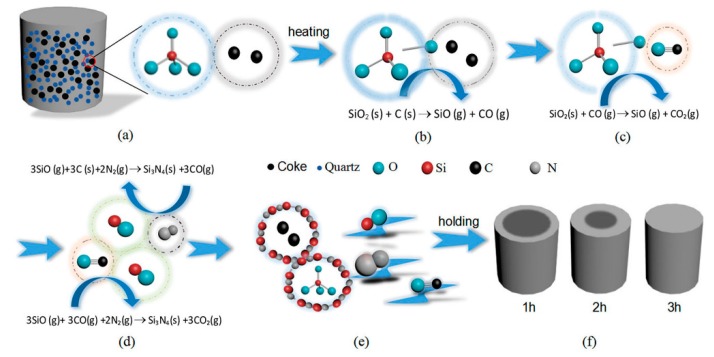
Reaction schematic diagram for the formation of β-Si_3_N_4_ by carbothermal reduction-nitridation (CRN) of quartz: (**a**) the green body of specimen, (**b**–**e**) nitridation processes of raw materials, (**f**) specimens obtained under different holding times.

Figure 5 is a schematic diagram demonstrating the synthesis of β-Si_3_N_4_ by CRN of quartz in two major steps. As the first step, illustrated by Figure 5a–c, SiO(g) forms via Equation (1) beyond certain temperature. Equation (1) requires direct contact of carbon and SiO_2_. Then in the next step, as shown in Figure 5d, Si_3_N_4_ nucleates from Equation (2), and grows up on the surface of carbon and SiO_2_, which is associated with the diffusion rate of SiO. The reaction process is progressive from exterior to interior of the sample pellets as shown in Figure 5f.

### 3.2. Influences of Starting Composition and Additive on Phase Composition and Morphology of Products

In order to elucidate the key factors for the synthesis of β-phase Si_3_N_4_ from quartz CRN synthesis, we investigated the effects of C/SiO_2_ molar ratio, Fe_2_O_3_ additive content, and the introduction of β-Si_3_N_4_ seeds on the products.

#### 3.2.1. Influence of C/SiO_2_ Molar Ratio on Phase Composition

According to Equation (5), the theoretical C/SiO_2_ molar ratio of Si_3_N_4_ is 2 [25]. Thus, samples S1–S4 with different C/SiO_2_ molar ratios (2, 2.2, 3, and 4) were designed and subjected to CRN synthesis at 1600 °C for 3 h.

3SiO_2_(s) + 6C(s) + 2N_2_(g) → Si_3_N_4_(s) + 6CO(g), *ΔG* = 1166.30–0.64T kJ/mol (5)

**Figure 6:**
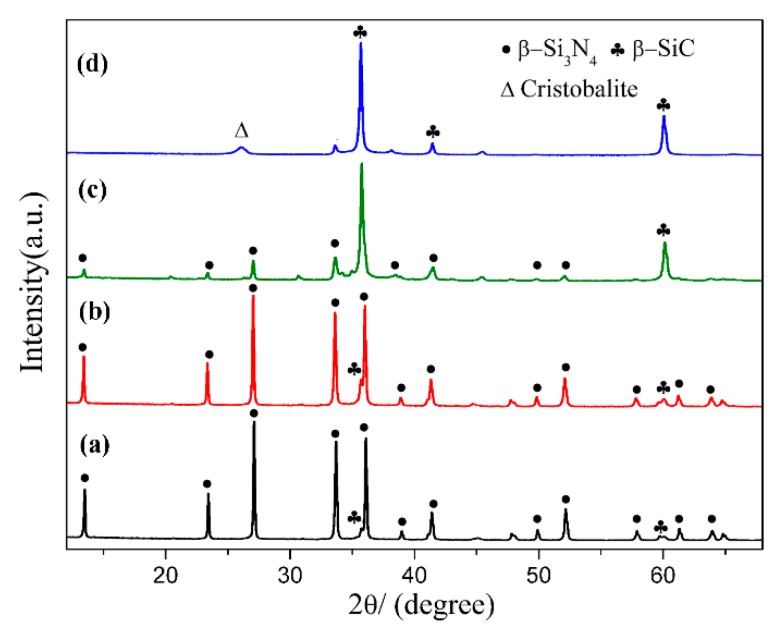
XRD patterns of sample S1~S4 nitrided at 1600 °C for 3 h: (**a**) S1, C/SiO_2_ = 2; (**b**) S2, C/SiO_2_ = 2.2; (**c**) S3, C/SiO_2_ = 3; (**d**) S4, C/SiO_2_ = 4.

Figure 6 shows the XRD patterns of samples S1~S4 nitrided at 1600 °C for 3 h. As seen in the figure, β-Si_3_N_4_ was the main phase when C/SiO_2_ was 2 (sample S1) and 2.2 (Sample S2), with trace amount of β-SiC. Peaks of β-Si_3_N_4_ decreased significantly when a higher C/SiO_2_ molar ratio of 3 was adopted in sample S3, and almost disappeared when the C/SiO_2_ ratio further increased to 4 (sample S4). At the meantime, β-cristobalite was detected which was derived from the phase transformation of residual quartz at high temperature. The results indicate that the theoretical/stoichiometric carbon content as per Equation (5) was optimal for preparing single-phase β-Si_3_N_4_ from quartz by CRN. Increased amount of β-SiC and other by-products formed in the product of samples with a higher C/SiO_2_ molar ratio.

#### 3.2.2. Influence of Fe_2_O_3_ Additive on Phase Composition and Morphology of Products

**Figure 7:**
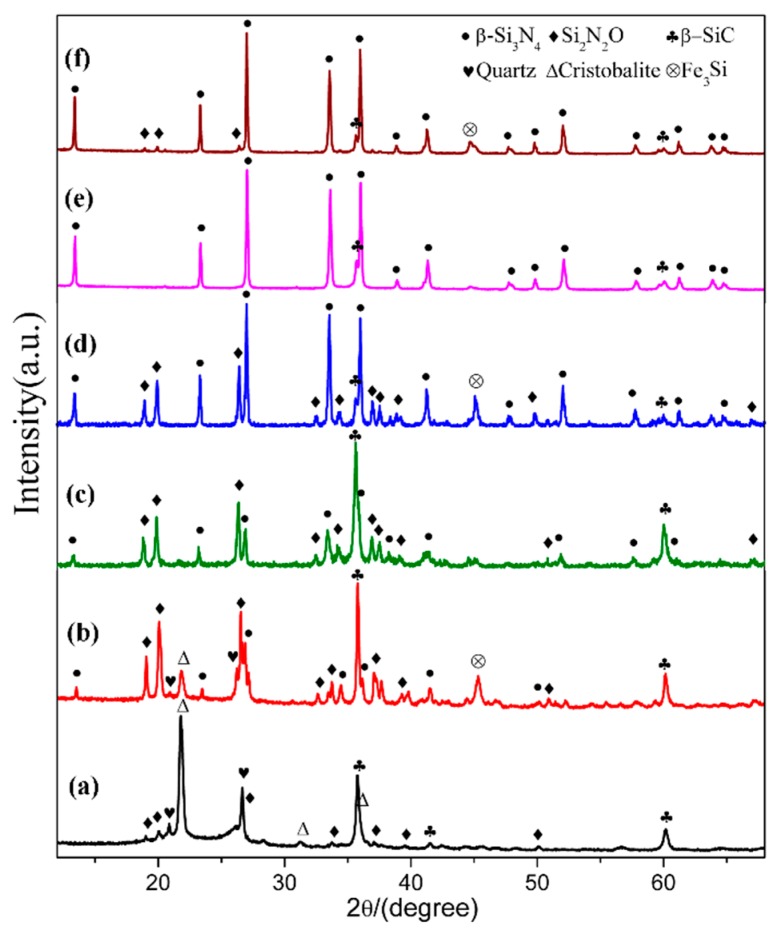
XRD patterns of sample S1(**a**,**c**,**e**), and sample S5 (**b**,**d**,**f**) at 1470, 1570, and 1600 °C, respectively.

In order to see the effect of additive, we designed a composition (sample S5) which added extra 4 wt.% Fe_2_O_3_ into the starting mixture. The XRD patterns of sample S1 (Fe_2_O_3_-free) and S5 (with extra 4 wt.% Fe_2_O_3_) are plotted in Figure 7, where the phase assemblages in the samples at different temperatures are compared. At 1470 °C, β-cristobalite, quartz and β-SiC were the main phases in sample S1. In contrast, much weaker peaks of cristobalite showed in sample S5 whereas Si_2_N_2_O and β-Si_3_N_4_ formed with a considerable amount. At 1570 °C, β-Si_3_N_4_, β-SiC and Si_2_N_2_O were present in both sample S1 and sample S5, while there was significantly more β-Si_3_N_4_ and much less β-SiC formed in sample S5 (comparing to S1). At 1600 °C, both samples had β-Si_3_N_4_ as dominant phase and trace amount of β-SiC, and the trace amount of Si_2_N_2_O were found in S5. Additionally, peaks appeared at 45.5° in sample S5 at all temperatures can be assigned to Fe_3_Si. The parameters and cell volumes of β-Si_3_N_4_ synthesized in S1 and S5 at 1600 °C (Figure 7e,f) are listed in Table 2. Table 3 is the weight fraction of each phase in S1 and S5 at 1570 and 1600 °C. These results illustrate that Fe_2_O_3_ could enhance the carbothermal reduction process of quartz and nitridation transformation to β-Si_3_N_4_.

**Figure 8:**
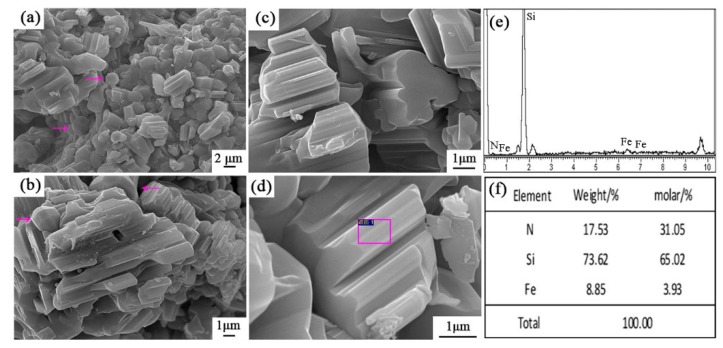
SEM images and EDS patterns of β-Si_3_N_4_ in sample S5 nitrided at 1600 °C: (**a**–**d**) SEM images with different magnification, (**e**,**f**) EDS results of the selected area in (**d**).

**Figure 9:**
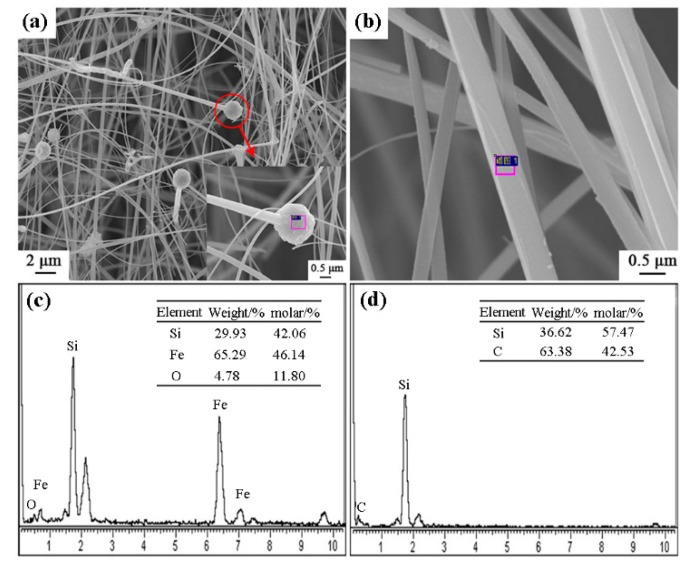
SEM images and EDS patterns of SiC fibers on the surface of sample S5 nitrided at 1600 °C: (**a**,**b**) SEM images of white-colored product layer, (**c**,**d**) EDS results of the selected area in (**a**,**b**).

Figure 8 shows the SEM images and EDS results of sample S5 nitrided at 1600 °C. As seen in Figure 8a–d, the columnar grains had gear-like morphologies and dominate in the products, which could be assigned to β-Si_3_N_4_ upon XRD and EDS results. A trace amount of Fe element was detected (Figure 8f), which was originated from the additive added in this sample. Furthermore, a white-colored product layer was covered on the surface of sample S5 nitrided at 1600 °C for 3 h. SEM observation shows that it was composed of fibers with widths of about 0.3–0.5 μm (Figure 9a,b). EDS result reveals that the fibers were SiC phase (Figure 9d). At the tip of each fiber, there was a spherical particle containing Si-Fe-O elements (Figure 9a,c). It is therefore suggested that the SiC fibers formed through a Vapor - Liquid - Solid (VLS) mechanism [26].

**Figure 10:**
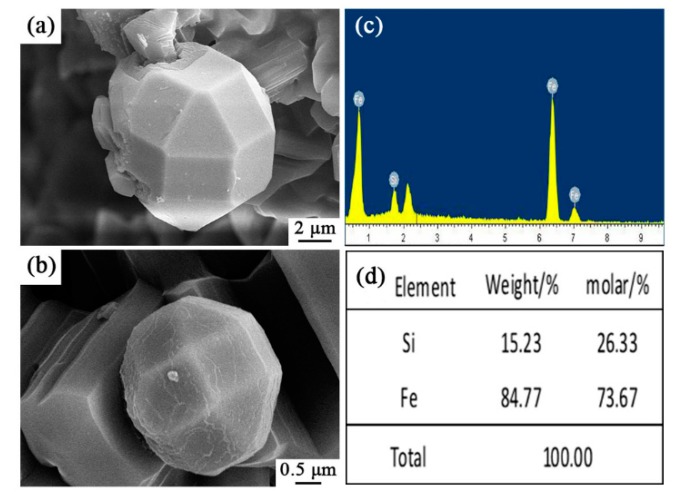
SEM images and EDS patterns of Si-Fe alloy of sample S5 nitrided at 1600 °C: (**a**,**b**) SEM images of spherical particles in sample S5, (**c**,**d**) EDS results of the selected area in (**a,b**).

Very interestingly, some spherical particles were observed in sample S5 (arrowed in Figure 8a,b), which were further characterized by SEM and EDS (Figure 10). It is wonderful to see that they were Archimedean solids with sizes of several microns. EDS results illustrate that they contained only Fe and Si elements with the molar ratio of Fe/Si ~2.79. This result clearly reveals that the faceted crystal was Fe_3_Si as detected in XRD data. To the best of our knowledge, such a novel morphology of Fe_3_Si was never reported in the literature.

Based on the above results, it can be concluded that Fe_2_O_3_ had a remarkable catalytic effect on the CRN of quartz at a lower temperature than 1600 °C. Under the reductive environment at high temperatures, carbon or CO can reduce Fe_2_O_3_ to Fe by Equations (6) or (7). It is known that the melting point of pure iron is 1538 °C. Therefore, Fe generally attaches to a support material in the reaction system by forming a liquid phase. In the present study, we speculate that Fe-Si-O liquid formed first (Equation (8), Figure 9). The Fe-containing liquid could dissolve SiO_2_ and C, then enhance their reaction to form SiO(g) [27] (Equation (9)), consequently promoting the formation of Si_2_N_2_O, SiC and even β-Si_3_N_4_ at lower temperature (Equations (3) and (4), Figure 7). Fe-containing liquid could significantly enhance the nitridation reaction, and the precipitation of β-Si_3_N_4_ has been known to be favored by the presence of Fe-containing liquid [28] (Equation (10)). Fe-Si-O liquids were further reduced to be Fe-Si containing liquid (Equation (11)). The Fe-rich liquid then crystallized as Fe_3_Si crystals while cooling (Equation (12)). The Fe-containing liquid phases, as a catalytic phase, promoted the formation of Si_3_N_4_, which is clearly illustrated by the phase assemblages in the samples at the low temperatures (Figure 5). Nevertheless, it is yet unclear how the unique Archimedean solids formed, which is beyond the objectives of this work and needs further investigation in near future.

Fe_2_O_3_(s) + 3C(s) → 2Fe(l) + 3CO(g), *ΔG* = 474.35–0.51T kJ/mol (6)

Fe_2_O_3_(s) + 3CO(g) → 2Fe(l)+3CO_2_(g), *ΔG*= −6.32–0.01T kJ/mol (7)

Fe (l) + SiO_2_(s) + C(s) → Fe-Si-O(l) + CO(g) (8)

(9) $${\mathrm{SiO}}_{2}\left(s\right)+C\left(s\right)\overset{Fe-Si-O\left(l\right)}{\to }\mathrm{SiO}\left(g\right)+\mathrm{CO}\left(g\right),\;\Delta G=665.59\;-\;0.33T\;\mathrm{kJ}/\mathrm{mol}\;$$

(10) $$3{\mathrm{SiO}}_{2}\left(s\right)+6C\left(s\right)+2N_{2}\left(g\right)\overset{Fe-Si-O(l)}{\to }{\mathrm{Si}}_{3}N_{4}\left(s\right)+6\mathrm{CO}\left(g\right),\;\Delta G\;=\;1166.30\;-\;0.64T\;\mathrm{kJ}/\mathrm{mol}$$

Fe-Si-O(l)+ C(s)/CO(g) → Fe-Si(l) + CO_2_(g) (11)

Fe-Si(l) → Fe_3_Si(s) (12)

#### 3.2.3. Influence of β-Si_3_N_4_ Seeds on Morphology of Products

**Figure 11:**
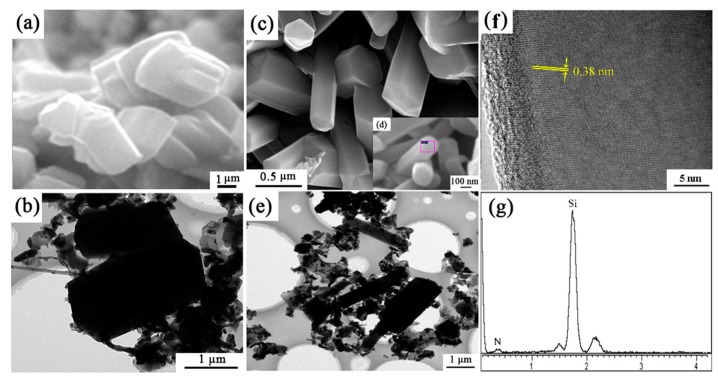
SEM, TEM/HRTEM images and EDS patterns of (**a**,**b**) sample S1 and (**c**–**g**) S6 nitrided at 1600 °C for 3 h.

The microstructure of sample S1 (no seeds) and S6 (with extra 2 wt.% Si_3_N_4_ seeds) synthesized at 1600 °C for 3 h were shown in Figure 11. In sample S1, the morphology of β-Si_3_N_4_ grains are short columnar, having grain sizes of around 3 μm. While in sample S6, grains were elongated and the gear-like morphology was rarely observed. From Figure 11c, the widths of the elongated grains are much smaller (0.2–0.5 µm) than those of short columnar grains in S1 (2–3 µm). HRTEM lattice image of the Si_3_N_4_ elongated grain indicates that the lattice fringe had no obvious distortion (Figure 11f,g). The measured lattice fringe spacing of 0.38 nm matched well with the (110) plane of β-Si_3_N_4_ (Figure 11f).

It is reported that introducing seeds into the starting materials could affect the microstructure of synthesized products and improve the physical properties of final ceramics [29,30]. For example, in the synthesis of Sialon powders, the addition of Sialon seeds would increase the growth competition among the grains, leading to an elongated columnar morphology, thus improving flexural strength and fracture toughness [24,31]. Herein, a significantly increased aspect ratio of Si_3_N_4_ grains was achieved by adding seeds into the starting mixtures. The addition of seeds could trigger heterogeneous nucleation [32]:

(13) $${\Delta G}^{*}=\frac{16\pi \sigma_{\alpha \beta }^{3}}{3\Delta g_{v_{v}}^{2}}f\left(\theta \right)={\Delta G}_{0}^{*}f\left(\theta \right)$$

where *θ* is contact angle between new Si_3_N_4_ nucleus and Si_3_N_4_ seeds, *f(θ)* is the influence function of *θ*, and ∆G* is Gibbs free energy of homogeneous nucleation. Therefore, when the contact angle was 0–180°, nucleation power could be reduced. Moreover, while crystal structure of seeds was the same as that of Si_3_N_4_, *θ* would be very small, which would reduce *∆G* and make more nucleus formed at the early stage of nitriding process. Thus, the growth competition between those nuclei would made the smaller and long columnization of Si_3_N_4_ grains.

### 3.3. The Possible Mechanisms of β-Si_3_N_4_ Single Polymorph Formation

Usually, α- and β- Si_3_N_4_ polymorphs are present in the products from carbothermal reduction nitridation of SiO_2_, and some related researches reported in the literature are summarized in Table 4. The phase composition ratio of α/β-Si_3_N_4_ in the CRN products was highly affected by additives (or impurities). For example, Sun et al. [8] reported that the yield of β-Si_3_N_4_ increased from 42.8% to 81.8% when improving CaF_2_ additive contents (Table 4 (1 and 2)). Wang et al. [22] revealed that the introduction of CaF_2_-Y_2_O_3_ additives significantly promoted the conversion of α-Si_3_N_4_ to β-Si_3_N_4_ (Tabele 4 (3 and 4)). Furthermore, the CRN temperature was another key factor. According to the literatures [33,34,35], the increasing CRN temperature was beneficial for the transformation of α-Si_3_N_4_ to β-Si_3_N_4_ (Table 4 (6 and 7), (8 and 9), (10 and 11)), and when it was higher than 1500 °C, β-Si_3_N_4_ emerged as the major phase, even single phase at 1700 °C (Table 4 (5)).

Herein, we successfully produced β-Si_3_N_4_ as the single polymorph (no α- phase co-exists), either with or without Fe_2_O_3_ as an additive. It is widely known that the α-Si_3_N_4_ derives from SiO vapor while β-Si_3_N_4_ from liquid phase during CNR process, and also there exist phase transformation between α-Si_3_N_4_ and β-Si_3_N_4_ [34,35]. As SiO vapor always forms during the reduction process, α-Si_3_N_4_ can form (temporarily at least) during the CRN process. In our study, the β-Si_3_N_4_ as the single polymorph formed regardless of adding Fe_2_O_3_ or not. Thus, we speculate the following three possible reasons that lead to the formation of single β-Si_3_N_4_ polymorph. Firstly, the silicon source used in this work was quartz mineral rather than commercial/pure amorphous or crystalline SiO_2_ powders in other literatures reported. The high temperature stability of quartz made nitriding formation process of Si_3_N_4_ took place at 1570 °C in catalyst-free sample. Secondly, according to the results and discussion above, α-Si_3_N_4_ was metastable at high temperature. α-phase might be generated in this work, but it was converted to β-phase at such high temperature. Last, the impurities in quartz raw material including 0.13 wt.% Fe_2_O_3_, or the formed eutectic liquid phases, contributed to the formation of pure β-phase in products, by either promoting the α-to-β phase transformation or direct formation of β-phase. The gear-like morphology of β-Si_3_N_4_ should be formed via coalescence in the iron-containing liquid phase.

### 3.4. PL Spectrum of Synthesized β-Si_3_N_4_ Samples

**Figure 12:**
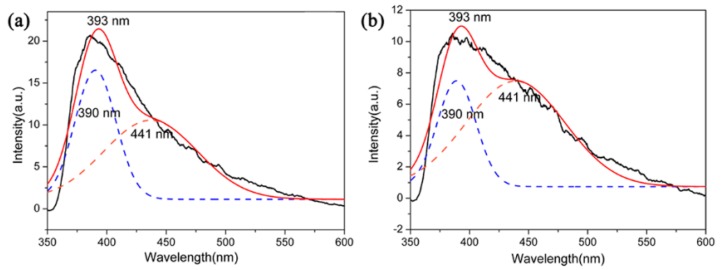
PL spectrum of (**a**) S5 and (**b**) S6 nitrided at 1600 °C.

The functional applications of Si_3_N_4_ had attracted increasing interest, for instance, the optical properties of Si_3_N_4_ films or nanostructures have been studied [36,37]. However, Si_3_N_4_ microcrystals had not attracted much attention in terms of their optical properties. In this context, PL emission properties (excitation at 330 nm) of β-Si_3_N_4_ microcrystals with different morphologies were tested by using samples S5 and S6 nitrided at 1600 °C. The PL spectra are showed in Figure 12. The spectrum of these two samples showed a board purple emission band with the maximum at 393 nm and it could be further fitted into two Guassion peaks 390 nm (3.17 eV) of purple spectral region and 441 nm (2.81 eV) of blue spectral region. It has been reported that as an indirect gap semiconductor, the luminescence of Si_3_N_4_ is attributed to a defect luminescence mechanism. The PL process of Si_3_N_4_ could be caused by the existing defect of it which including ≡Si–Si≡, =N^−^, ≡Si^0^ and ≡Si^−^, and those were corresponding to four types of defects of Si–Si bond, N–N bond, and Si–N dangling bonds [38,39]. The experimental data may shed light on potential application of Si_3_N_4_ microcrystals in optical devices.

## 4. Conclusions

A carbothermal reduction-nitridation (CRN) method was used for the preparation of β-Si_3_N_4_ powders from natural quartz and coke powders. β-Si_3_N_4_ powders with relatively high purity were obtained at 1600 °C for 3 h, with trace amount of β-SiC formed as a by-product. The results indicated that β-Si_3_N_4_ powders were obtained in the products, accompanied by the appearance of β-SiC and Si_2_N_2_O during the CRN process. The temperature, holding time, C/SiO_2_ malor ratio, Fe_2_O_3_ addition, and β-Si_3_N_4_ seeds played important roles for the synthesis β-Si_3_N_4_ powders. The optimal nitriding temperature was 1600 °C and holding time was 3 h. A stoichiometric molar ratio of C/SiO_2_ of 2 was preferred for preparing β-Si_3_N_4_ with better phase purity. Fe_2_O_3_ under the reducing environment formed Fe-containing liquid phases, which promoted the reaction thermodynamics of the CRN process. The products were mainly gear-like β-Si_3_N_4_ grains and Fe_3_Si Archimedean solids. On the other hand, adding β-Si_3_N_4_ seeds into the starting mixture led to elongated β-Si_3_N_4_ grains with much finer widths and big specific ratio. The as-synthesized Si_3_N_4_ microcrystals exhibited an intense violet‒blue spectral range with two maximum peaks at 441 nm (3.17 eV) and 390 nm (2.81 eV), which may shed light on potential application of Si_3_N_4_ microcrystals in optical devices.

## Figures and Tables

**Table 1:** Compositional design of the samples (wt.%).

Samples	C/SiO_2_ Molar Ratio	Coke	Quartz	Fe_2_O_3_ (Extra)	β-Si_3_N_4_ (Extra)
S1	2	30.77	69.23	0	0
S2	2.2	32.84	67.16	0	0
S3	3	40.00	60.00	0	0
S4	4	47.06	52.94	0	0
S5	2	30.77	69.23	4	0
S6	2	30.77	69.23	0	2

**Table 2:** Lattice parameters and cell volume of β-Si_3_N_4_ in S1 & S5.

Samples	Littice Parameters		Cell Volume(Å^3^)
	a(Å) = b(Å)	c(Å)	
S1, 1570 °C, Figures 2e and 7c	7.6351	2.8867	145.7343
S1, 1600 °C, Figures 2f and 7e	7.8607	2.9139	155.9293
S5, 1570 °C, Figure 7d	7.7205	2.8695	148.1248
S5, 1600 °C, Figure 7f	7.7753	2.8993	151.7952

**Table 3:** Weight fraction of each phase in S1 and S5 at 1570 and 1600 °C (wt. %).

Samples	β-Si_3_N_4_	SiC	Si_2_N_2_O	Fe_3_Si
S1, 1570 °C, Figure 2e and Figure 7c	32.20	36.06	31.74	0
S1, 1600 °C, Figure 2f and Figure 7e	91.52	8.48	0	0
S5, 1570 °C, Figure 7d	57.46	13.41	20.74	9.39
S5, 1600 °C, Figure 7f	85.47	6.85	4.22	3.46

**Table 4:** Comparison of reaction conditions and products by carbothermal reduction nitridation of SiO_2_ reported in the literature and the present study.

No.	Raw materials	Carbon Source	Additive	T/°C-t/h	Products	Reference
1	Amorphous SiO_2_	carbon black	CaF_2_-1%	1500-2	β(42.8%) and α	[8]
2	Amorphous SiO_2_	carbon black	CaF_2_-10%	1500-2	β(81.8%) and α	[8]
3	Amorphous SiO_2_	carbon black	None	1500-2	β(52.4%) and α	[22]
4	Amorphous SiO_2_	carbon black	CaF_2_, Y_2_O_3_	1500-2	β(90%) and α	[22]
5	Amorphous SiO_2_	carbon black	CaF_2_, Y_2_O_3_	1700-2	β(100%)	[22]
6	Quartz powder	carbon black	Y_2_O_3_, Bentonite	1450-3	β(26.9%), α	[34]
7	Quartz powder	carbon black	Y_2_O_3_, Bentonite	1500-3	β(85.7%), α	[34]
8	Synthetic silica	activated charcoal	MgO or Y_2_O_3_	1375-3	Si_2_N_2_O, β:α = 1:9	[35]
9	Synthetic silica	activated charcoal	MgO or Y_2_O_3_	1475-3	β:α = 1:2	[35]
10	Quartz sand	activated charcoal	Fe(NO_3_)_3_	1300-3	quartz, Si_2_N_2_O, β:α = 3:4	[36]
11	Quartz sand	activated charcoal	Fe(NO_3_)_3_	1540-3	β:α = 9:1	[36]
12	Quartz	coke powders	None	1470-3	cristobalite, SiC and Si_2_N_2_O	This article
13	Quartz	coke powders	Fe_2_O_3_	1470-3	SiC, Si_2_N_2_O, β, quartz	This article
14	Quartz	coke powders	None	1600-3	β(100%)	This article

Note: α, β: Si_3_N_4._

## References

[1] Li T.F., Chen Y.J., Li W., Li J.B., Luo L.J., Yang T., Liu L.Y., Wu G.L. (2018). Fabrication and mechanical properties of boron nitride nanotube reinforced silicon nitride ceramics. Ceram. Int..

[2] Huang J.T., Zhang S.W., Huang Z.H., Liu Y.G., Fang M.H. (2013). Growth of α-Si_3_N_4_ nanobelts via Ni-catalyzed thermal chemical vapour deposition and their violet-blue luminescent properties. CrystEngComm.

[3] Hu X., Shao C.W., Wang J., Wang H., Cheng J. (2017). Effects of residual radicals on compositional and structural stability of silicon nitride fibers. J. Eur. Ceram. Soc..

[4] Wu J.M., Zhang X.Y., Xu J., Gan K., Li J.L., Li C.H., Yang J.L., Shi Y.S. (2015). Preparation of porous Si_3_N_4_ ceramics via tailoring solid loading of Si_3_N_4_ slurry and Si_3_N_4_ poly-hollow microsphere content. J. Adv. Ceram..

[5] Liu X.Z., Yi X.M., Guo R., Li Q.D., Nomura T. (2017). Formation mechanisms of Si_3_N_4_ microstructures during silicon powder nitridation. Ceram. Int..

[6] Park Y.J., Park M.J., Kim J.M., Lee J.W., Ko J.W., Kim H.D. (2014). Sintered reaction-bonded silicon nitrides with high thermal conductivity: The effect of the starting Si powder and Si_3_N_4_ diluents. J. Eur. Ceram. Soc..

[7] Strong K.T., Arreguin S.A., Bordia R.K. (2016). Controlled atmosphere pyrolysis of polyureasilazane for tailored volume fraction Si_3_N_4_/SiC nanocomposites powders. J. Eur. Ceram. Soc..

[8] Sun S.Y., Wang Q., Ge Y.Y., Tian Z.B., Zhang J., Xie Z.P. (2018). Synthesis of well-dispersed columnar Si_3_N_4_ using carbothermal reduction–nitridation method. Powder Technol..

[9] Karakuş N., Kurt A.O., Duran C., Öztürk C., Toplan H.Ö. (2013). Sintering behaviour of silicon nitride powders produced by carbothermal reduction and nitridation. Adv. Powder Technol..

[10] Magnani G., Galvagno S., Sico G., Portofino S., Freda C., Burresi E. (2016). Sintering and mechanical properties of β-SiC powder obtained from waste tires. J. Adv. Ceram..

[11] Yin L., Xu Y.G., Huang Z.H., Liu Y.G., Fang M.H., Liu B.L. (2013). Synthesis of ZrN–Si_3_N_4_ composite powders from zircon and quartz by carbothermal reduction and nitridation. Powder Technol..

[12] Arik H. (2003). Synthesis of Si_3_N_4_ by the carbo-thermal reduction and nitridation of diatomite. J. Eur. Ceram. Soc..

[13] Anggraini L., Natsume Y., Ameyama K. (2016). Effect of particle shape on dispersion formation of harmonic microstructure of Si_3_N_4_-ZrO_2_. Mater. Sci. Forum.

[14] Hu Z.L., Zhu T.B., Wu W.W., Peng Z.J., Hu F., Xie Z.P. (2018). Growth mechanism of α-Si_3_N_4_ submicron rods prepared from amorphous Si_3_N_4_ powders. Ceram. Int..

[15] Yu J.J., Guo W.M., Wei W.X., Lin H.T., Wang C.Y. (2018). Fabrication and wear behaviors of graded Si_3_N_4_ ceramics by the combination of two-step sintering and β-Si_3_N_4_ seeds. J. Eur. Ceram. Soc..

[16] Huang J.T., Huang Z.H., Yi S., Liu Y.G., Fang M.H., Zhang S.W. (2013). Fe-catalyzed growth of one-dimensional α-Si_3_N_4_ nanostructures and their cathodoluminescence properties. Sci. Rep..

[17] Li B., Li G.Q., Chen J.H., Chen H.Y., Xing X.M., Hou X.M., Li Y. (2018). Formation mechanism of elongated β-Si_3_N_4_ crystals in Fe-Si_3_N_4_ composite via flash combustion. Ceram. Int..

[18] Chen K., Huang Z.H., Liu Y.G., Fang M.H., Huang J.T., Xu Y.G. (2013). Synthesis of β-Si_3_N_4_ powder from quartz via carbothermal reduction nitridion. Powder Technol..

[19] Dahal N., Chikan V. (2010). Phase-controlled synthesis of iron silicide (Fe_3_Si and FeSi_2_) nanoparticles in solution. Chem. Mater..

[20] Ortega A., Alcalá M.D., Real C. (2008). Carbothermal synthesis of silicon nitride (Si_3_N_4_): Kinetics and diffusion mechanism. J. Mater. Process. Technol..

[21] Li J., Shao G., Ma Y., Zhao X.T., Wang H.L., Zhang R. (2019). Processing and properties of polycrystalline cubic boron nitride reinforced by SiC whiskers. Int. J. Appl. Ceram. Technol..

[22] Wang Q., Sun S.Y., Li S., Guo Z.Y. (2018). Carbothermal synthesis of approximately spherical Si_3_N_4_ particles with homogeneous size distribution. Ceram. Int..

[23] Ji H.P., Huang Z.H., Chen K., Li W.J., Gao Y.F., Fang M.H., Liu Y.G., Wu X.W. (2014). Synthesis of Si_3_N_4_ powder with tunable α/β-Si_3_N_4_ content from waste silica fume using carbothermal reduction nitridation. Powder Technol..

[24] Huang J.T., Miao Y.P., Zhang M., Feng Z.J., Hu Z.H., Li X.B., Luo J.M. (2018). Hot-pressed sintered Ca-α-Sialon ceramics with grains from short prismatic to elongated morphology synthesized via carbothermal reduction and nitridation. J. Alloy. Compd..

[25] Li X.M., Li R., Zhu X.T., Zhou Y.L., Ren G.N., Zhang L. (2017). Properties of large-sized porous Si_3_N_4_ ceramic tubes fabricated by carbothermal reduction of diatomite preforms. Ceram. Int..

[26] Charoo M.S., Wani M.F. (2016). Friction and wear properties of nano-Si_3_N_4_/nano-SiC composite under nanolubricated conditions. J. Adv. Ceram..

[27] Li X.L., Wang J., Ji H.M., Xu X.W. (2012). Catalytic Effect and Mechanism of Fe_2_O_3_ on Synthesis of Si_2_N_2_O by Carbothermal Reduction and Nitridation of SiO_2_. Aerosp. Mater. Technol..

[28] Boyer S.M., Moulson A.J. (1978). A mechanism for the nitridation of Fe-contaminated silicon. J. Mater. Sci..

[29] Guo W.M., Yu J.J., Xiong M., Wu S.H., Lin H.T. (2016). High-toughness Lu_2_O_3_-doped Si_3_N_4_ ceramics by seeding. Ceram. Int..

[30] Wang B., Yang J., Guo R. (2009). Microstructure and property enhancement of silicon nitride-barium aluminum silicate composites with β-Si_3_N_4_ seed addition. J. Mater. Sci..

[31] Marin E., Adachi T., Boschetto F. (2018). Biological response of human osteosarcoma cells to Si_3_N_4_-doped Bioglasses. Mater. Des..

[32] Jun Y.S., Kim D., Neil C.W. (2016). Heterogeneous nucleation and growth of nanoparticles at environmental interfaces. Accounts Chem. Res..

[33] Topateş G. (2018). Direct production of Si_3_N_4_ foams by carbothermal reduction and nitridation of SiO_2_. Ceram. Int..

[34] Karakus N., Kurt A.O., Toplan H.Ö. (2009). Synthesizing high α-phase Si_3_N_4_ powders containing sintering additives. Ceram. Int..

[35] Bandyopadhyay S., Mukerji J. (1991). Reaction sequences in the synthesis of silicon nitride from quartz. Ceram. Int..

[36] Wang F., Qin X.F., Yang L.X. (2015). Synthesis and photoluminescence of Si_3_N_4_ nanowires from La/SiO_2_ composites and Si powders. Ceram. Int..

[37] Huang J.T., Zhang S.W., Huang Z.H. (2012). Catalyst-assisted synthesis and growth mechanism of ultra-long single crystal α-Si_3_N_4_ nanobelts with strong violet-blue luminescent properties. CrystEngComm.

[38] Robertson J., Powell M.J. (1984). Gap states in silicon nitride. Appl. Phys. Lett..

[39] Xiong L., Dai J.H., Song Y. (2018). Effects of doping on photoelectrical properties of one-dimensional α-Si_3_N_4_ nanomaterials: A first-principles study. Phys. B Condens. Matter.

